# Geospatial tools in leprosy elimination: Enhancing precision in active case detection and resource allocation

**DOI:** 10.1371/journal.pdig.0001068

**Published:** 2025-11-07

**Authors:** Anil Fastenau, Denis A. Yawovi Gadah, Akila Wimima Bakoubayi, Piham Gnossike, Felicitas Schwermann, Matthew Willis, Fabian Schlumberger, Thomas Hambridge, Sundeep Chaitanya Vedithi, Sophie C. W. Stuetzle, Patricia D. Deps, Nimer Ortuño-Gutiérrez

**Affiliations:** 1 Marie Adelaide Leprosy Center, Karachi, Pakistan; 2 German Leprosy and Tuberculosis Relief Association (DAHW), Wuerzburg, Germany; 3 Department of Global Health, Institute of Public Health and Nursing Research, University of Bremen, Bremen, Germany; 4 Heidelberg Institute of Global Health (HIGH), University of Heidelberg, Heidelberg, Germany; 5 German Leprosy and Tuberculosis Association, Lomé, Togo; 6 Department of Public Health, Faculty of Health Sciences, University of Lomé, Lomé, Togo; 7 National Program for Neglected Tropical Diseases, Lomé, Togo; 8 School of Medicine, Dentistry & Biomedical Sciences, Queen’s University Belfast, Belfast, United Kingdom of Great Britain and Northern Ireland; 9 Erasmus MC, University Medical Centre Rotterdam, Rotterdam, The Netherlands; 10 Department of Medicine, University of Cambridge, Cambridge, United Kingdom of Great Britain and Northern Ireland; 11 Department of Social Medicine, Federal University of Espírito Santo, Vitória, Brazil; 12 Damien Foundation, Brussels, Belgium; Massachusetts Institute of Technology, UNITED STATES OF AMERICA

## Background

Leprosy, known also as Hansen’s disease, is an infectious highly stigmatizing neglected tropical disease (NTD) that may cause permanent disabilities [1]. Despite significant advances in treatment and control, leprosy remains a global public health concern with close to 200,000 new cases of leprosy notified worldwide annually [2]. Reaching out to missing and hidden cases, along with early diagnosis, are priorities for successful leprosy control and elimination [3]. Geographical Information Systems (GIS) have shown promising results in improving disease control strategies by identifying high-risk areas for targeted interventions [4]. Additionally, GIS provides the ability to display spatial distribution of diseases by integrating geographical data with tabular information from sources such as spreadsheets, tables, and graphs [5]. We advocate for concrete integration of GIS in leprosy control, aiming at the elimination of transmission, underscoring its potential to refine active case detection (ACD), optimize resource allocation, and enhance cost-effectiveness

## Geographical information systems: A key to cost-effective leprosy control and elimination

Efficient resource use is a key challenge in leprosy control and elimination, as traditional ACD methods are resource-intensive and hard to scale in constrained regions. GIS mapping offers a solution by providing accurate, real-time data on the spatial distribution of leprosy cases [6]. By identifying geographic hotspots, GIS enables health authorities to focus their interventions where they are most needed, thereby maximizing the cost-effectiveness of leprosy elimination efforts [7].

In Brazil, GIS-driven campaigns targeting high-incidence areas led to the detection of 50% of all new leprosy cases during one intervention, demonstrating how spatial mapping can significantly improve the outcomes of active case-finding efforts [8]. In low-endemic settings, the ability to direct interventions toward areas of greatest need can dramatically enhance the efficiency of leprosy control and elimination programs, reducing costs and ensuring better coverage [9].

## Geographical information systems and geospatial analysis in active case detection

In addition to cost-effectiveness, GIS and cluster analysis can guide decisions on the most appropriate ACD methods for different regions [10]. Leprosy is known to cluster at various geographic levels, from household contacts to broader community areas [11]. By applying geospatial and cluster analysis techniques, public health authorities can determine the most effective strategies for each context. For instance, in Bihar, India, spatial analysis at the hamlet level revealed clusters of new leprosy cases that can be targeted for active screening and post-exposure prophylaxis (PEP) [4]. In high-endemic settings like the Comoros and Madagascar, geospatial analysis highlighted the need to extend case-finding efforts to entire communities where over 50% of the population resides near an index case [12]. This approach has proven effective in detecting hidden cases and interrupting transmission, further emphasizing the role of GIS in refining and improving leprosy control and elimination strategies [13].

## Designing interventions with geographical information systems for limited-resource settings

**Fig 1:**
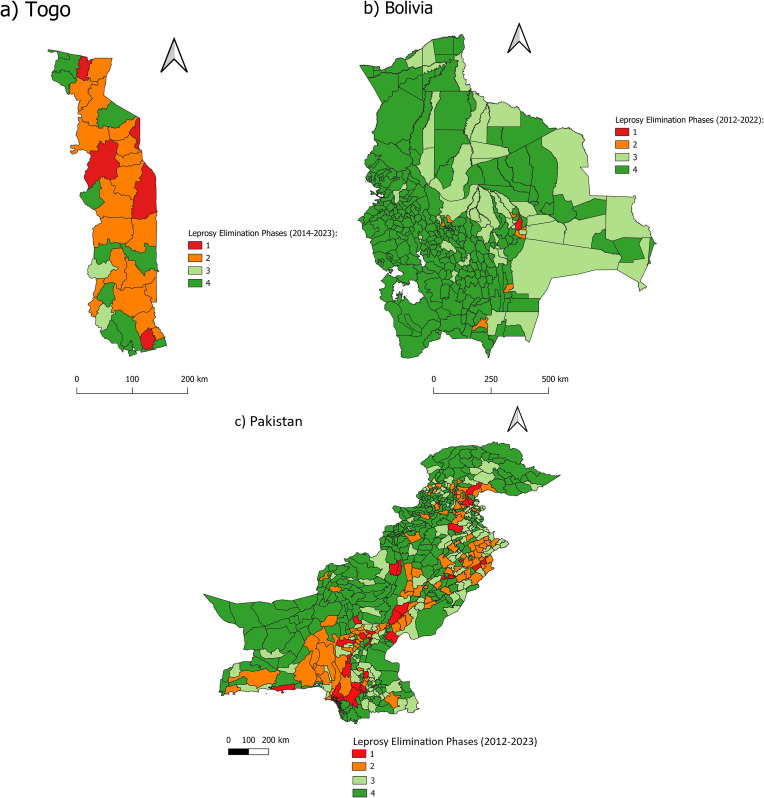
Leprosy Elimination Monitoring Tool in (a) Togo, (b) Bolivia, and (c) Pakistan at the municipality level. Phase 1: Until interruption of transmission (No new autochthonous cases among children for at least 5 consecutive years); Phase 2: interruption of transmission until elimination of disease (No new autochthonous cases for at least 3 consecutive years and no child cases in 5 years); Phase 3: Post-elimination surveillance (No or only sporadic autochthonous cases for a period of ≥10 years); Phase 4. Non-endemic status: leprosy is not normally present in the area or country. Sporadic cases may occur.

For countries with limited resources, GIS offers a powerful tool for optimizing both financial and human capital needed for effective leprosy control and elimination. By identifying precise geographic areas of high transmission, GIS allows public health programs to deploy resources where they will have the greatest impact. In Pakistan, Togo, and Bolivia (low endemic for leprosy) [14] an initial step was the identification of phase-1 (ongoing transmission) using the Leprosy Elimination Monitoring Tool (LEMT) for improving SDR-PEP implementation, case detection, and treatment outreach efforts (Fig 1).

Moreover, GIS can help determine the most appropriate methods for ACD, which may vary significantly between regions [10]. For example, while door-to-door screenings may be necessary in certain high-endemic areas [15], contact tracing efforts may suffice in regions with lower transmission rates [14].

## Recommendations for policy and practice

To fully integrate GIS in leprosy control and elimination, national and international health authorities must commit to integrating these technologies into public health programs, especially in resource-constrained settings. Key recommendations include:

1. Incorporate GIS into monitoring and evaluation of National Leprosy Programs or National NTD Programs: investment in GIS technologies enables targeted interventions and reduces unnecessary expenditures. Smartphones and open-source apps are less expensive and have acceptable precision in recording GPS coordinates and ensure confidentiality of data recorded.
2. Use Geospatial and Cluster Analysis to Guide Interventions: By leveraging spatial data, health authorities can determine which active case-finding strategies are most appropriate for different geographic and epidemiological contexts, ensuring cost-effectiveness.
3. Train Local Health Workers in GIS Tools: Empowering health workers with GIS technology will enable them to take ownership of data collection and analysis, improving the overall precision of leprosy control and elimination efforts. Training should be focused on practical and standardized procedures of data displaying, cluster analysis, and surveillance with open-source and user-friendly software.
4. Safeguard Data Privacy through Clear Governance: Since leprosy is highly stigmatized, GIS applications should protect confidentiality by working with de-identified or aggregated data and by ensuring that any geolocation information collected is handled responsibly. Where cloud-based or third-party systems are used, data ownership, storage, and legal jurisdiction must be defined through clear governance frameworks.
5. Expand the integration of GIS Beyond Leprosy in other health programs: While GIS has proven highly effective in leprosy control, its application should extend to other NTDs such as leishmaniasis and poverty-related diseases such as tuberculosis, particularly in low-resource settings and under-represented regions. Mapping multiple diseases simultaneously can allow for more integrated, multi-disease intervention strategies.

## Conclusion

GIS represents a set of complementary tools, with the LEMT providing a simple, widely applicable entry point using routine district-level data. The LEMT is feasible for use in all countries where leprosy is still endemic and can reveal both high-burden districts and areas of ongoing transmission. Where technical and financial capacity allows, more advanced GIS applications can add precision by identifying clusters at finer scales. The key principle is that every country should apply the level of GIS that is practical and sustainable. Used in this way, GIS becomes a powerful instrument for public health decision making, helping to focus activities on the most vulnerable populations, improve cost-effectiveness, strengthen active case detection, and guide the allocation of scarce resources. As global efforts advance towards zero leprosy, embedding GIS and spatial analysis within leprosy control and elimination strategies will be essential to achieve lasting impact.
